# Balloon Uncrossable Left Main Coronary Artery Dissection: A Case Report and Literature Review

**DOI:** 10.1002/ccr3.71353

**Published:** 2025-10-28

**Authors:** Syed Yaseen Naqvi, Ahmed Qasim Alhaideri, Ali Razzaq, Syed Mohammad Naqvi

**Affiliations:** ^1^ Department of Internal Medicine, College of Medicine University of Warith Al‐Anbiyaa Karbala Iraq; ^2^ University of Limerick Limerick Ireland; ^3^ Alkafeel Hospital Karbala Iraq

**Keywords:** balloon‐uncrossable lesion, calcified coronary lesion, left main coronary artery dissection, percutaneous coronary intervention

## Abstract

Coronary artery dissection presents a high‐risk scenario, particularly when complicated by severe calcification. We report a complex case of a balloon‐uncrossable lesion encountered during percutaneous coronary intervention (PCI) of a calcified ostial‐to‐mid left anterior descending (LAD) artery lesion extending into the LMCA. A 54‐year‐old male with type 2 diabetes and exertional angina underwent coronary angiography revealing severe calcified LAD disease. Despite successful initial balloon pre‐dilatation, a drug‐eluting stent (DES) could not cross the lesion. Withdrawal attempts resulted in a large LMCA‐to‐LAD dissection flap, preventing further device passage. Urgent multidisciplinary consultation was initiated. A novel approach using forceful advancement of a 1.0 mm semi‐compliant balloon during deflation enabled successful lesion crossing. This was followed by lesion preparation, overlapping DES deployment, and optimized post‐dilatation. The patient remained stable, with excellent angiographic results. This case underscores the importance of innovation and teamwork in managing complex, calcified coronary lesions with unexpected procedural complications.

**Summary**

- Management of calcified Type C lesions requires advanced tools and a multidisciplinary heart team approach.
- Innovative bailout maneuvers can facilitate device delivery but should be limited to experienced operators.
- Optimal post‐stent results depend on precise optimization using POT, kissing balloon techniques and use of intracoronary imaging.

## 1 Introduction

Left main coronary artery (LMCA) disease is a critical condition associated with high morbidity and mortality. Percutaneous coronary intervention (PCI) for LMCA lesions, especially in calcified or complex anatomies, poses a significant challenge. In this case, we describe a unique complication encountered during PCI of a severely calcified left anterior descending (LAD) lesion extending into the LMCA, leading to an uncrossable balloon situation, and its successful management with innovative interventional techniques.

## 2 Case History/Examination

A 54‐year‐old male with a history of type 2 diabetes mellitus (T2DM) and dyslipidemia presented with class III exertional angina. He had no history of hypertension or a family history of premature cardiovascular disease (CVD). His resting ECG was normal, and echocardiography showed grade 1 diastolic dysfunction with an ejection fraction (EF) of 63%. A treadmill stress test was positive for inducible ischemia, prompting coronary angiography.

## 3 Investigations and Treatment

**FIGURE 1:**
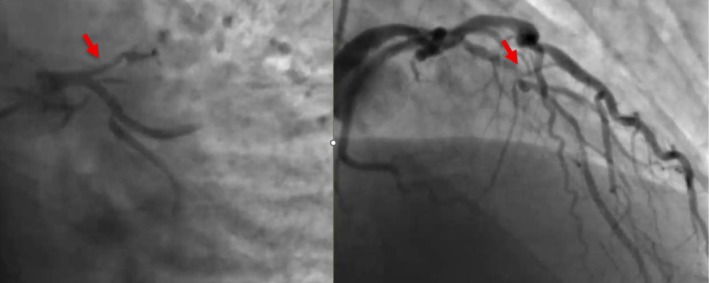
Coronary angiography revealed unobstructed left main coronary artery (LMCA), severe ostial to mid calcified long segment left anterior descending (LAD) artery disease.

**FIGURE 2:**
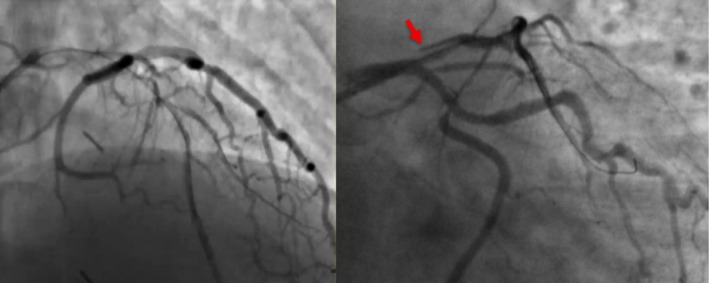
Coronary angiography revealed a large distal LMCA to ostial LAD calcified dissection flap, preventing further passage of any balloon or device despite good guide support and buddy wire placement.

**FIGURE 3:**
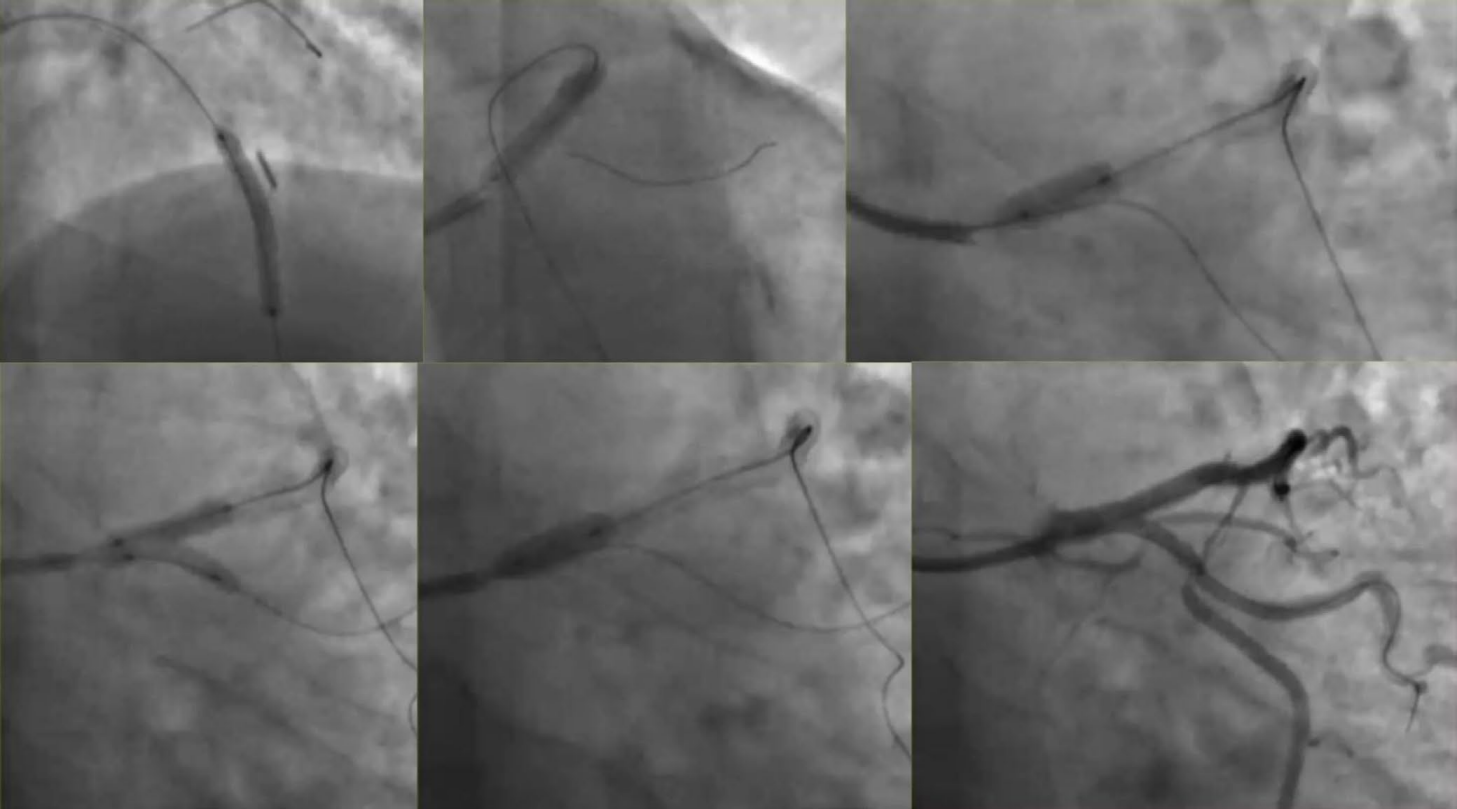
Coronary angiography showing the steps involved to complete the PCI with two overlapping DES to cover the entire lesion. Post‐dilatation techniques were employed, including proximal optimization technique (POT), kissing balloon inflation, and final POT.

**FIGURE 4:**
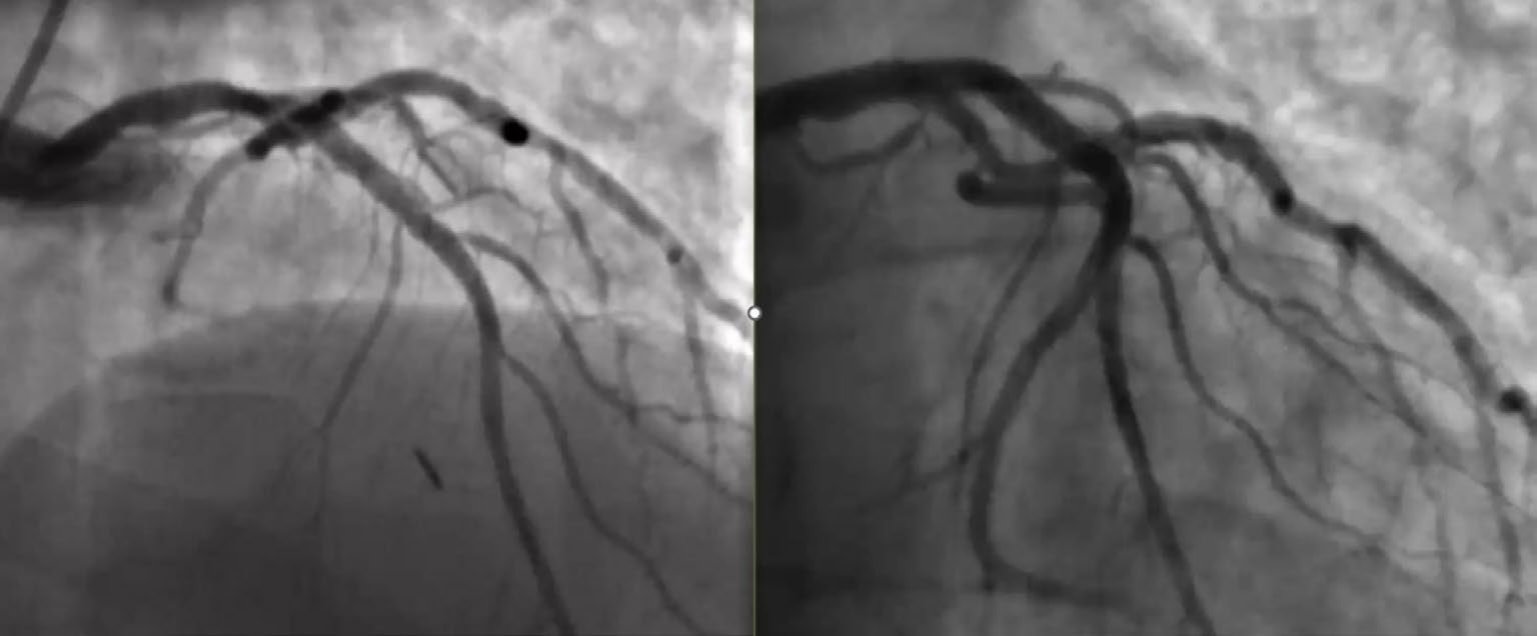
Coronary angiography showing an excellent angiographic outcome with TIMI 3 flow.

His coronary angiogram revealed unobstructed left main coronary artery (LMCA), severe ostial to mid calcified long segment left anterior descending (LAD) artery disease, mild non‐obstructive left circumflex disease, and severe proximal to mid calcified right coronary artery (RCA) disease (Figure 1). Heart team discussion was done and the patient opted for PCI. The patient was pretreated with clopidogrel and the decision was made to perform PCI to the LAD initially, with a plan for RCA PCI in a later session. The procedure was performed via the right radial approach using a 6French sheath. The LMCA was intubated with a 6F EBU guide catheter, and two workhorse wires were placed in the distal LAD and diagonal branch. Balloon pre‐dilatation was performed in the ostial to mid LAD using a 1.5 × 20 mm and 2.5 × 20 mm semi‐compliant balloons, which crossed the lesion successfully and were inflated at high pressure. After lesion preparation, a 2.75 × 26 mm drug‐eluting stent (DES) could not cross the proximal LAD lesion. At the time it was felt to be secondary to significant lesion calcification. On withdrawal of the stent for further lesion preparation, no balloon could cross the lesion. Angiography revealed a large LMCA to ostial LAD calcified dissection flap, preventing further passage of any balloon or device despite good guide support and buddy wire placement (Figure 2). The heart team was alerted, and a multidisciplinary discussion ensued, including a cardiac surgeon and a second experienced interventional cardiologist. We did not have the availability of a debulking device in our lab; therefore while the operating room was being prepared, multiple attempts were made to introduce a small balloon for re‐entry and high‐pressure inflation, but all failed. A novel technique was employed where a small 1.0 × 10 mm semi‐compliant Ryurei balloon (Terumo) was advanced to the site where forward progression had stopped. The balloon was inflated at 10 atm and was forcefully introduced during balloon deflation. This maneuver successfully allowed the balloon to cross the calcified dissection flap. Larger balloons were then used for further lesion preparation. Two overlapping DES were deployed to cover the entire lesion, including the LMCA shaft. Gold standard post‐dilatation techniques were employed, including proximal optimization technique (POT), kissing balloon inflation, and final POT, achieving an excellent angiographic outcome (Figures 3 and 4) (Video S1).

## 4 Conclusion and Results

The patient remained hemodynamically stable throughout the procedure. Final angiography confirmed successful stent deployment with restored coronary flow and sealing of the dissection. The patient was monitored postoperatively in the coronary care unit. He was discharged home the following day on dual antiplatelet therapy (DAPT) and optimal medical therapy. One week later, the patient underwent an uneventful and successful PCI of his RCA.

## 5 Discussion

This case highlights the substantial challenges involved in percutaneous coronary intervention (PCI) for severely calcified lesions, particularly the risk of dissection during balloon or stent manipulation. Balloon‐uncrossable lesions represent a significant obstacle, especially in chronic total occlusions (CTOs) or cases involving tortuous, calcified coronary arteries. In most cases, lesion resistance is evident from the outset. However, in our case, the lesion was initially balloon‐crossable during pre‐dilatation, but became balloon‐uncrossable when stent delivery was attempted, underscoring the dynamic nature of lesion behavior during intervention.

To improve success rates in such scenarios, several escalation strategies can be employed. These include optimizing guide catheter support, using specialized low‐profile balloons, guide catheter extensions, wire‐based support techniques, anchoring maneuvers, and microcatheters for lesion preparation and device delivery [1]. When first‐line approaches fail, advanced lesion modification techniques such as balloon‐assisted microdissection, excimer laser coronary atherectomy (ELCA), and high‐speed rotational atherectomy (HSRA) are often necessary [2].

Guide catheter support is critical in managing balloon‐uncrossable lesions. Both passive and active support can be enhanced through the use of large‐caliber catheters (7F or 8F) with appropriate shapes that provide better coaxial alignment and engage the contralateral aortic wall for added backup. Deep engagement further increases catheter stability but comes with risks such as coronary dissection and damping [3]. In complex interventions, particularly CTOs, operators frequently begin with larger catheters to ensure sufficient support for crossing and device delivery [4].

Lesion preparation typically involves noncompliant balloons matched 1:1 to vessel diameter. Operators often begin with low‐profile, semi‐compliant balloons featuring hydrophilic coatings, such as the Sapphire Pro, Mini‐Trek, Sprinter Legend, Across CTO, Apex Push, and Chocolate balloons, which are designed for tight lesion negotiation [5]. The novel Blimp balloon (Abbott Vascular), an ultralow‐profile design, has demonstrated potential in facilitating CTO PCI but remains under investigation [6]. Oscillating the balloon during advancement can reduce friction and help navigate through resistant segments [7]. The method used in our case of forceful balloon advancement during balloon deflation has not been previously described in the literature. The main risk with employing our methods for lesion crossing is causing further extension of the coronary dissection and loss of wire position. It is difficult to quantify the exact force used to cross the lesion during balloon deflation, but we recommend applying similar force to delivering any standard balloon but only during balloon deflation. We believe that the lesion will actively modify its structure during balloon deflation thereby allowing successful balloon crossing.

Guide catheter extensions—including devices such as the Heartrail II, Guideliner, Guidezilla, and Telescope—allow for deep intubation into tortuous or calcified vessels, significantly enhancing support and improving pushability [8]. These extensions have been associated with high procedural success, with reports showing a 93% lesion‐crossing rate using the Guideliner in complex cases [9]. Adjunctive techniques such as the “inchworm” method, in which a balloon is inflated beyond the extension tip and deflated to advance the extender, can facilitate deeper engagement of the extension system [10].

Wire‐based strategies also contribute significantly to procedural success. The Wiggle wire, with multiple bends along its shaft, redistributes forces to navigate resistant segments. The buddy wire technique, involving a second wire to straighten the vessel and reduce system friction, is widely utilized in difficult anatomies [11]. The wire‐cutting technique, which entails balloon inflation over a second wire followed by rapid wire withdrawal, creates controlled trauma in the lesion to allow subsequent balloon passage [12].

Anchoring techniques provide additional stability by inflating a balloon in a side branch proximal to the target lesion, thereby increasing guide catheter support. Correct balloon sizing is essential to avoid complications such as dissection or perforation [13]. Alternatively, a side‐branch wire anchor can offer modest additional support. The combination of a guide catheter extension with an anchor balloon further augments support and facilitates lesion crossing in resistant cases [14].

Microcatheters, including the Tornus, Corsair, Finecross, Turnpike, and Mamba, offer varying levels of flexibility, torque control, and penetrative force to aid in crossing balloon‐uncrossable lesions [15]. The Tornus, with its metal coil construction, is particularly suited for penetrating heavily calcified segments, while the Corsair's hybrid design makes it valuable in both antegrade and retrograde CTO interventions [16].

When conventional devices fail to cross the lesion, second‐line techniques are considered. Balloon‐assisted microdissection, which involves intentional balloon rupture against the lesion to create microdissections, can facilitate device passage or subintimal access. Although relatively simple and cost‐effective, its success rate is approximately 50%, and the technique carries risks such as vessel staining or perforation [17, 18].

ELCA, which delivers ultraviolet laser pulses via a catheter to modify plaque through photothermal, photochemical, and photomechanical mechanisms, can facilitate plaque debulking and device delivery. Laser fluences range from 30 to 80 mJ/mm^2^ with pulse rates of 25–80 Hz. Use in contrast media enhances efficacy but also increases perforation risk [19]. ELCA is particularly useful in lesions resistant to balloon inflation, especially when combined with other plaque modification strategies [20].

HSRA utilizes the ROTABLATOR or ROTAPRO system with burr sizes ranging from 1.25 to 2.5 mm. It modifies calcified plaque by ablating superficial calcium and creating microfractures, thus enabling subsequent balloon expansion and stent delivery [21]. A limitation of HSRA is the need for a specialized 0.009″ guidewire, which may not be advanceable through uncrossable lesions. Orbital atherectomy (OA), though less commonly available in Europe, provides another effective option for modifying calcified lesions, particularly when traditional approaches are unsuccessful [22]. Unfortunately, we did not have the availability of rotational atherectomy in our lab; therefore these techniques were not performed.

In some cases, combining ELCA and HSRA—referred to as RASER angioplasty—can be employed. ELCA is first used to create a small pilot channel, enabling the RotaWire to pass through the lesion and allowing successful execution of HSRA [23]. This staged approach has proven valuable in severely calcified, balloon‐uncrossable lesions.

While current European Society of Cardiology (ESC) guidelines recommend intravascular imaging with IVUS or OCT to optimize PCI outcomes in complex lesions, these modalities were not available in our case [24]. We believe the use of intravascular imaging would have identified and quantified the calcium burden in more detail, thereby potentially modifying our PCI strategy, perhaps using upfront rotational atherectomy.

In summary, balloon uncrossable lesions require a stepwise approach utilizing guide catheter support, specialized balloon and wire techniques, and advanced debulking strategies. When first‐line methods fail, techniques such as balloon‐assisted microdissection, ELCA, and HSRA improve procedural success. The selection of appropriate tools and techniques tailored to individual lesion characteristics remains critical in optimizing PCI outcomes. In our case, the balloon uncrossable lesion was successfully crossed using a small‐profile balloon which was advanced during balloon deflation.

## Author Contributions

**Syed Yaseen Naqvi:** conceptualization, supervision, validation, writing – original draft, writing – review and editing. **Ahmed Qasim Alhaideri:** writing – review and editing. **Ali Razzaq:** writing – review and editing. **Syed Mohammad Naqvi:** supervision, writing – original draft, writing – review and editing, writing – review and editing.

## Ethics Statement

This case report was conducted in accordance with institutional ethical standards and the Declaration of Helsinki.

## Consent

Written informed consent was obtained from the patient for publication of the clinical details and accompanying images.

## Conflicts of Interest

The authors declare no conflicts of interest.

## Supporting information


**Video S1:** Coronary angiography loops of the procedure showing initial baseline diagnostic angiogram, left main into LAD dissection flap and final result after DES ×2 placement.

## Data Availability

The data that supports the findings of this study are available in the Supporting Information of this article.
