# Supplementary figures and images for: Balloon Uncrossable Left Main Coronary Artery Dissection: A Case Report and Literature Review
Source: Clin Case Rep. 2025 Oct 28;13(11):e71353. doi: 10.1002/ccr3.71353 (PMC12568374; doi:10.1002/ccr3.71353)

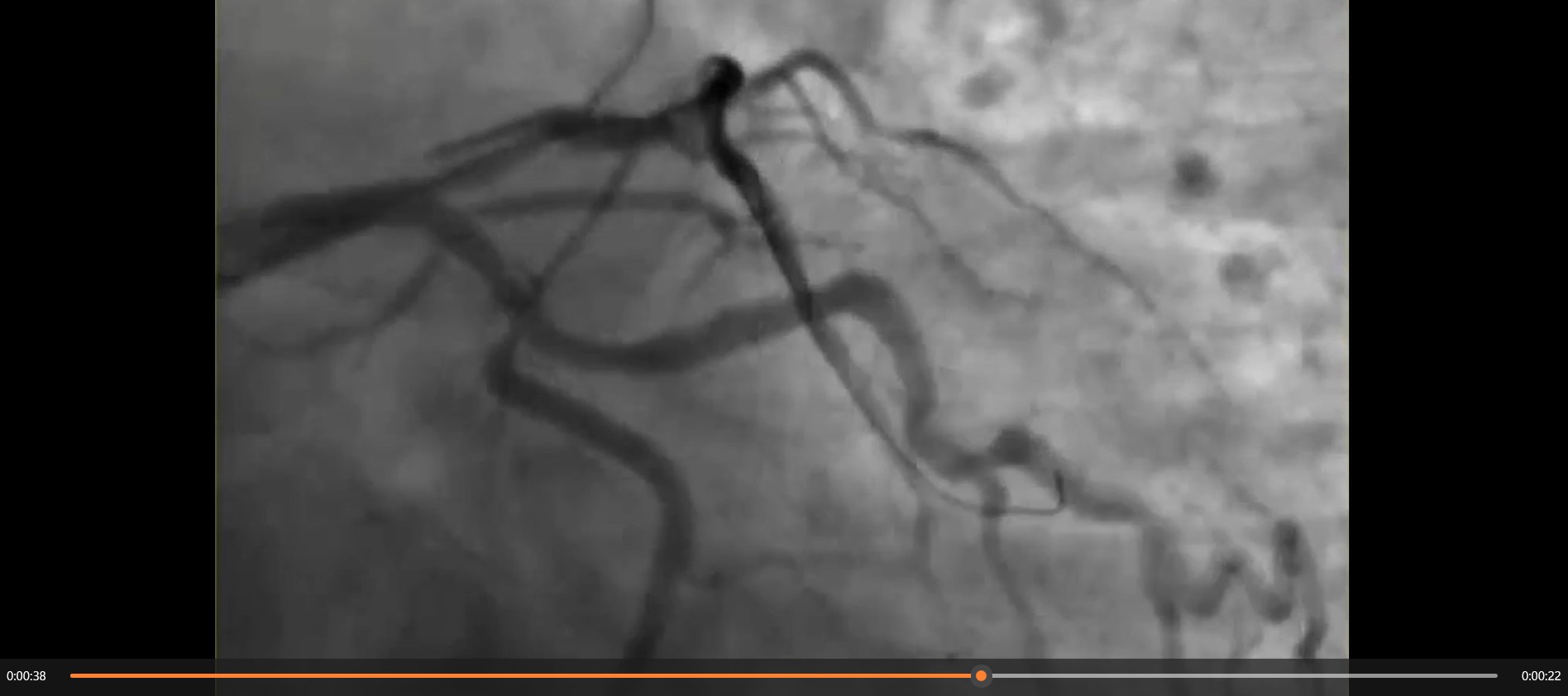

Supplement: Supplementary file 1 — Video S1: Coronary angiography loops of the procedure showing initial baseline diagnostic angiogram, left main into LAD dissection flap and final result after DES ×2 placement. [file CCR3-13-e71353-s001.zip › ccr371353-sup-0003-Supinfo3@Place holder image for video.jpg]
